# Reducing the burden of inconclusive smart device single-lead ECG tracings via a novel artificial intelligence algorithm

**DOI:** 10.1016/j.cvdhj.2023.12.003

**Published:** 2023-12-27

**Authors:** Simon Weidlich, Diego Mannhart, Alan Kennedy, Peter Doggart, Teodor Serban, Sven Knecht, Jeanne Du Fay de Lavallaz, Michael Kühne, Christian Sticherling, Patrick Badertscher

**Affiliations:** ∗ University Hospital Basel, Basel, Switzerland; † Cardiovascular Research Institute Basel (CRIB), Basel, Switzerland; ‡ PulseAI, Belfast, United Kingdom

**Keywords:** Smart device, Smartwatch, Artificial intelligence, Single-lead electrocardiogram, Atrial fibrillation, Digital health

## Abstract

**Background:**

Multiple smart devices capable of automatically detecting atrial fibrillation (AF) based on single-lead electrocardiograms (SL-ECG) are presently available. The rate of inconclusive tracings by manufacturers’ algorithms is currently too high to be clinically useful.

**Method:**

This is a prospective, observational study enrolling patients presenting to a cardiology service at a tertiary referral center. We assessed the clinical value of applying a smart device artificial intelligence (AI)-based algorithm for detecting AF from 4 commercially available smart devices (AliveCor KardiaMobile, Apple Watch 6, Fitbit Sense, and Samsung Galaxy Watch3). Patients underwent a nearly simultaneous 12-lead ECG and 4 smart device SL-ECGs. The novel AI algorithm (PulseAI, Belfast, United Kingdom) was compared with each manufacturer’s algorithm.

**Results:**

We enrolled 206 patients (31% female, median age 64 years). AF was present in 60 patients (29%). Sensitivity and specificity for the detection of AF by the novel AI algorithm vs manufacturer algorithm were 88% vs 81% (*P* = .34) and 97% vs 77% (*P* < .001) for the AliveCor KardiaMobile, 86% vs 81% (*P* = .45) and 95% vs 83% (*P* < .001) for the Apple Watch 6, 91% vs 67% (*P* < .01) and 94% vs 82% (*P* < .001) for the Fitbit Sense, and 86% vs 82% (*P* = .63) and 94% vs 80% (*P* < .001) for the Samsung Galaxy Watch3, respectively. In addition, the proportion of SL-ECGs with an inconclusive diagnosis (1.2%) was significantly lower for all smart devices using the AI-based algorithm compared to manufacturer’s algorithms (14%–17%), *P* < .001.

**Conclusion:**

A novel AI algorithm reduced the rate of inconclusive SL-ECG diagnosis massively while maintaining sensitivity and improving the specificity compared to the manufacturers’ algorithms.

## Introduction

Smart devices are increasingly used, both in the general population and in clinical medicine. Smart devices are FDA cleared (U.S. Food and Drug Administration) and CE marked (Conformité Européenne), confirming the safety and quality standard, including the 30-second single-lead ECG (SL-ECG) recording. Therefore, they are approved for the diagnosis of atrial fibrillation (AF) according to the latest European Society of Cardiology guidelines for AF diagnosis.^1^ Although manual confirmation of an AF diagnosis is required, multiple smart devices are equipped with automated rhythm classification using artificial intelligence (AI). Those rhythm classifications consist of sinus rhythm (SR), AF, bradycardia (heart frequency <50 beats/min), tachycardia (heart frequency >100–150 beats/min), inconclusive, and poor recording.

However, automated rhythm interpretation is conservative, since a false-positive diagnosis could have severe consequences. Thus, many automated diagnoses from SL-ECGs are deemed inconclusive despite yielding a readable single-lead ECG tracing. Manual clinician interpretation of even a fraction of potential inconclusive tracings will further burden an already overloaded medical system, contradicting the original purpose of this technology, especially since it is estimated that in 2023 the number of smartwatch users is exceeding 200 million.^2^

The accuracy of different smart devices has been assessed in numerous studies,^3^, ^4^, ^5^ demonstrating a rate of inconclusive tracings between 18% and 31%.^3^^,^^4^ Automated algorithms using AI have previously been successfully used to analyze photoplethysmography-based recordings as well as Holter ECGs, non–commercially available wearable ECG recordings, or implantable loop recorder ECGs.^6^, ^7^, ^8^, ^9^, ^10^ However, limited experience exists regarding the application of an AI algorithm for single-lead ECG classification recorded from different smart devices. To overcome this challenge, we hypothesized that a novel, commercially available AI algorithm (PulseAI; PulseAI Ltd, Belfast, United Kingdom)^11^^,^^12^ allows accurate rhythm detection with fewer inconclusive classifications and with similar or better sensitivity and specificity compared to the automated manufacturer-based SL-ECG classification from different smart devices.

## Methods

### Study design and population

This is a prospective, single-center diagnostic study enrolling patients presenting to a tertiary hospital for electrophysiological interventions (the University Hospital of Basel, Switzerland) between April 2021 and September 2022. Patients included were admitted for electric cardioversions, pacemaker or implantable cardioverter-defibrillator implantation, or catheter ablation procedures and had to be aged >18 years to participate in this study. Excluded were patients with missing recordings or logistical or technical issues. The study was approved by the local ethics committee, was preregistered (ClinicalTrials.gov, NCT04809922), and was conducted according to the principles of the Declaration of Helsinki. Informed consent was provided by all patients included in the study. The design, data collection, and analysis were conducted according to the STROBE guidelines for observational studies^13^ (Supplemental Table 1). All authors vouch for the data and analysis, drafted the paper together, and made the decision to submit for publication.

### Study aims and assessment

The goal of this study was to determine if using a novel AI algorithm can improve the accuracy of automated SL-ECG rhythm classification and reduce the number of inconclusive tracings compared to the manufacturer’s algorithm. Once enrolled, patients underwent a preprocedural 12-lead ECG (part of routine clinical care) followed by 5 wearable smart device recordings for 30 seconds (AliveCor KardiaMobile or KardiaMobile 6L, Fitbit Sense, Samsung Galaxy Watch 3, or Withings ScanWatch). The 12-lead ECGs were obtained with a standard ECG recorder (Schiller SDS-200, Touch 4.4.3; Schiller, Baar, Switzerland) with a sweep speed of 25 mm/s and a standard augmentation of 10 mm/mV. SL-ECGs from the smart devices were generated and viewed according to instructions provided by the manufacturers. The SL-ECGs from the above-mentioned smart devices were exported as vectorized PDF files. Vectorized PDF ECGs contain more information and are better recognized by computer systems than rasterized PDF ECGs and are therefore better suited for precise rhythm classification. The study compared the diagnostic accuracy for detecting AF using the novel AI algorithm and the manufacturer’s algorithm. Five commercially available wearable smart devices as mentioned above were used for recording. Each SL-ECG was 30 seconds long and recorded consecutively, while the patient was sitting at rest at a table for an optimal setting. SL-ECGs generated by the Withings ScanWatch were excluded, since the PDF exports were rasterized and not vectorized. All manufacturer-based algorithms automatically interpret a heart rhythm below 50 or above 100–150 beats/min (depending on the manufacturer) as inconclusive. The study used a physician-interpreted 12-lead ECG, which was recorded within minutes before the smart device SL-ECGs, as the gold standard to compare rhythm classification with the smart device–recorded tracings.

### ECG interpretation

#### Physicians’ interpretation

Each 12-lead ECG and the corresponding SL-ECGs of the same patient were exported as PDF files, anonymized, and distributed to 2 blinded cardiologists. Each tracing was evaluated independently, and rhythm was determined as SR, AF, or inconclusive. Disagreements between the 2 cardiologists’ initial diagnoses were reviewed and assessed by a third cardiologist. In case of disagreement, the diagnosis of the third cardiologist was considered as the definitive diagnosis.

All inconclusive tracings (except for bradycardia and tachycardia) were reviewed by 2 cardiologists and classified according to predefined categories possibly causing an inconclusive rhythm classification. These categories were conduction delay, artefact, ectopy, and ventricular pacing. Conduction delay was defined as QRS width ≥120 ms. Ectopy encompassed premature atrial or ventricular contractions. Artifacts were defined as baseline shifts, noise, or no identifiable QRS complexes for at least 2 R-R intervals. These anomalies were chosen based on previous experience as well as documented and reported possible pitfalls in generating a conclusive diagnosis^3^^,^^14^^,^^15^ and furthermore were chosen by the authors based on the PulseAI’s diagnostic abilities.

#### AI interpretation

The PulseAI Deep Neural Network (PDNN)^11^^,^^12^ takes as an input the raw ECG data (sampled at 256 Hz, or 256 samples per second) in microvolts and outputs a single multiclass prediction of the cardiac rhythm. The PDNN is similar to the standard residual network architecture used by Ribeiro and colleagues,^16^ commonly used for computer vision applications but adapted to 1-dimensional signals. The PDNN has 9 convolutional layers, consisting of 4 residual blocks with 2 convolutional layers per block. Each residual block performs down-sampling via max pooling. We applied batch normalization, rectified linear activation, and dropout to help with regularization. The final layer is a fully connected layer with a sigmoid activation, producing a multiclass probability of each ECG rhythm.

The PDNN was trained de novo with random initialization of the weights. We used the Adam optimizer and a mini-batch size of 32. We initialized the learning rate (0.001) and reduced it by a factor of 10 when the testing set loss stopped improving for 2 consecutive epochs. During PDNN training, the weights were altered to minimize differences between the PDNN’s output and the reference annotations. We trained the PDNN on a randomly selected single lead from the 12-lead signal for each training mini-batch to maximize the exposure of the network to different waveform morphologies and amplitudes, allowing for better generalizability. By not relying on training data exclusively from smart devices, the neural network remains unbiased and can effectively process and interpret ECG signals from many sources. The training process was repeated for all ECGs in the training set, consisting of more than 1 million ECGs from a private internal database. This database employed for developing the neural network underwent a stringent anonymization process right from its source and had undergone prior assessment by experienced emergency medicine physicians and cardiologists. The ECGs used in this study were collected from various sources, encompassing regions such as the United States, Europe, and Asia. Each ECG underwent a thorough examination by healthcare professionals as part of standard clinical care procedures. The AF prevalence of the training set was 6%. To ensure the reliability and accuracy of the neural network, a separate dataset from smart devices was incorporated solely for validation purposes. It is noteworthy that no smart device ECG recordings were included in the neural network’s training database, ensuring an unbiased evaluation of its performance during the subsequent validation phase.

For this study for the purpose of direct comparison, the PulseAI algorithm was set to interpret tracings with the same possible outcomes as the manufacturer’s algorithms. Therefore, SL-ECGs were interpreted as either SR, AF, or inconclusive, in cases where no diagnosis could be made.

By incorporating data from diverse sources and adhering to rigorous quality control measures, this study ensured the representativeness and generalizability of the neural network’s performance, which is not limited to a specific type or brand of smart device. Including globally obtained ECGs and the rigorous oversight by healthcare professionals during the data collection process enhances the robustness and reliability of the network’s outcomes. The network was trained end-to-end with no ECG preprocessing steps.

### Statistical methods

Continuous variables were reported as either mean and standard deviation (SD) or median (interquartile range), as appropriate, while categorical variables were presented as numbers and percentages. For comparisons between the rate of inconclusive tracings, the Fisher exact test was used. We calculated and compared the sensitivity, specificity, and positive predictive value (PPV) of the AF detection algorithm diagnoses (manufacturer and novel AI) using the cardiac electrophysiologist–interpreted 12-lead ECG as the reference standard. To compare the 2 algorithms, we used the McNemar test for values that are paired. We included valid inconclusive results to prevent misleading conclusions regarding the accuracy and clinical usefulness of smart devices. Therefore, inconclusive results were counted as false-positives or false-negatives, depending on the underlying rhythm determined by the cardiologists. All statistical analyses were performed using RStudio version 2022.07.2.

## Results

### Baseline data

Of the 258 patients, 52 were excluded owing to missing SL-ECGs, leaving 206 patients in the final cohort. Therefore 206 SL-ECGs were generated by each of the 4 devices included in the study, resulting in a total of 824 SL-ECGs for further analysis. The mean age was 64 years (SD ±13 years); 31% of patients were female. AF was present in 60 patients (29%) during the time of recording.

### Reducing the rate of inconclusive tracings

**Figure 1:**
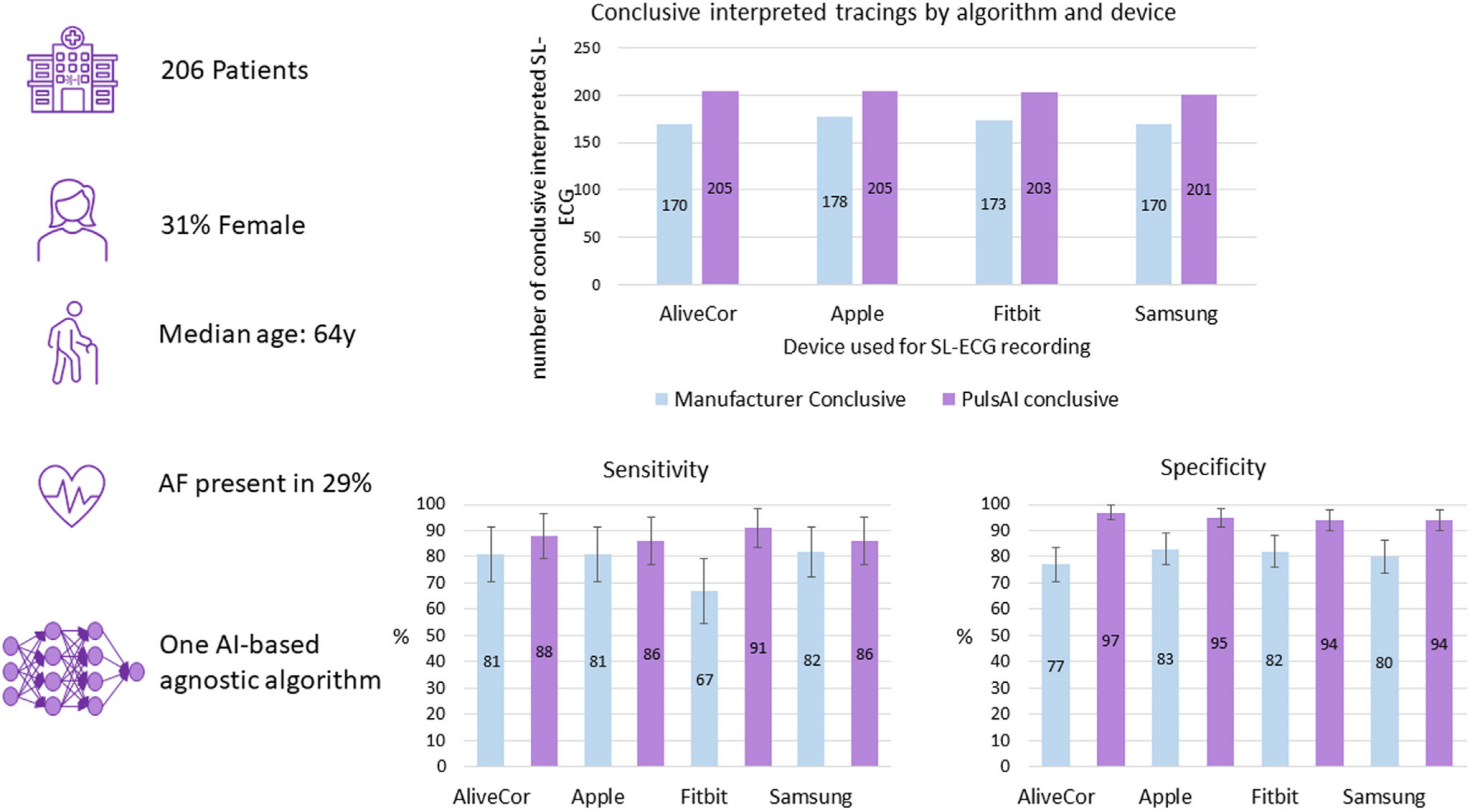
Sensitivity and specificity for detecting atrial fibrillation by the novel artificial intelligence (AI) algorithm vs manufacturer algorithm are depicted for each assessed smart device (bottom). The proportion of single-lead electrocardiograms with a conclusive diagnosis is illustrated for the novel AI algorithm vs manufacturer algorithm for each assessed smart device (top). AF= atrial fibrillation; SL-ECG = single-lead electrocardiogram.

Across all 824 SL-ECGs, 133 (16%) SL-ECGs were labeled as inconclusive by at least 1 smart device, compared to 10 (1.2%) inconclusive SL-ECGs by the AI algorithm (*P* < .001) (Figure 1). From the 206 SL-ECG tracings recorded by each device, AliveCor labeled 36 (17%), Apple 28 (14%), Fitbit 33 (16%), and Samsung 36 (17%) SL-ECGs as inconclusive, vs 1 (<1%), 1 (<1%), 3 (1%), and 5 (2%) inconclusive from the AI algorithm (*P* < .001).

### Reasons for inconclusive tracings

In Table 1 we provide categories with possible reasons for inconclusive tracings. Although there were differences in tachycardia classification, possibly related to different heart rate cut-offs, there were no differences in conduction delay, artifact, ectopy, and ventricular pacing possibly causing inconclusive SL-ECGs between different smart devices. An adjudicated number of artifact-corrupted data with both a conclusive and inconclusive interpretation for the manufacturer’s algorithms as well as for the PulseAI algorithm can be found in Supplemental Table 2.

**Table 1:** Comparison between algorithms and manual interpretation regarding anomalies in the inconclusive cases

	AliveCor algorithm	AliveCor with PulseAI algorithm	Apple algorithm	Apple with PulseAI algorithm	Fitbit algorithm	Fitbit with PulseAI algorithm	Samsung algorithm	Samsung with PulseAI algorithm
Total inconclusive SL tracings (out of 206 individual tracings per device)	36 (17.5%)	1 (0.5%)	28 (13.6%	1 (0.5%)	33 (16.1%)	3 (1.5%)	36 (17.5%)	5 (2.4%)
Reason for SL-ECG anomalies in inconclusive-labeled SL-ECGs								
Bradycardia	0	0	1 (3.6%)	0	7 (21%)	0	6 (17%)	0
Tachycardia	3 (8.3%)	0	2 (7.1%)	0	11 (33%)	0	5 (14%)	0
Artifact	15 (42%)	0	17 (61%)	0	8 (24%)	3 (100%)	17 (47%)	0
Ectopy	7 (19%)	0	12 (43%)	0	8 (24%)	0	5 (14%)	1 (20%)
Ventricular conduction delay	11 (31%)	1 (100%)	9 (32%)	1 (100%)	9 (27%)	3 (100%)	8 (22%)	1 (20%)
Ventricular pacing	10 (28%)	1 (100%)	7 (25%)	1 (100%)	7 (21%)	2 (67%)	6 (17%)	2 (40%)

Multiple anomalies are possible for each SL-ECG.

SL = single-lead; SL-ECG = single-lead electrocardiogram.

### Comparing sensitivity, specificity, and PPV

Overall, the novel AI algorithm had a sensitivity of 88% (confidence interval [CI] 83%–92%), a specificity of 95% (CI 93%–97%), and a PPV of 87% (CI 83%–92%) (Figure 1). Although the novel AI algorithm showed a significant improvement in specificity for detecting AF compared to each manufacturer-based algorithm, sensitivity only increased in 1 case. For AliveCor, the AI algorithm achieved a specificity of 97% and sensitivity of 88%, whereas the manufacturer’s algorithm had a specificity of 77% (*P* < .001) and sensitivity of 81% (*P* = .34). For Apple Watch, the AI algorithm achieved a specificity of 95% and sensitivity of 86%, whereas the manufacturer’s algorithm had a specificity of 83% (*P* < .001) and sensitivity of 81% (*P* = .45). For Fitbit, the AI algorithm achieved a specificity of 94% and sensitivity of 91%, whereas the manufacturer’s algorithm had a specificity of 82% (*P* < .001) and sensitivity of 67% (*P* < .001). For the Samsung Watch, the AI algorithm achieved a specificity of 94% and sensitivity of 86%, whereas the manufacturer’s algorithm had a specificity of 80% (*P* < .001) and sensitivity of 82% (*P* = .63).

### Comparing AI vs manual interpretation

Across all smart devices, manual interpretation of the 824 SL-ECGs by the 3 cardiologists, compared with the 12-lead ECG as gold standard, had a sensitivity of 98% (CI 96%–100%), a specificity of 97% (CI 95%–98%), and a PPV of 93% (CI 89%–96%). Sensitivity, specificity, and PPV of manual interpretation for each smart device are listed in Table 2 and were in the range of the novel AI algorithm. Interestingly, in 3 out of the 10 SL-ECGs labeled inconclusive by the AI algorithm (30%), the first 2 cardiologists disagreed with each other’s rhythm classification.

**Table 2:** Comparison between algorithms and manual interpretation of all 206 individual single-lead electrocardiograms by each device

Smart device	Manufacturer’s algorithm			Novel AI algorithm			Manual interpretation		
	Sensitivity	Specificity	PPV	Sensitivity	Specificity	PPV	Sensitivity	Specificity	PPV
AliveCor KardiaMobile	81% (70-91%)	77% (70-83%)	57% (46-68%)	88% (79-96%)	97% (94-100%)	91% (83-99%)	98% (91-100%)	97% (92-99%)	92% (82-97%)
Apple Watch 6	81% (70-91%)	83% (77-89%)	65% (54-76%)	86% (77-95%)	95% (92-99%)	88% (79-96%)	98% (91-100%)	97% (93-99%)	94% (84-98%)
Fitbit Sense	67% (54-79%)	82% (76-88%)	58% (46-70%)	91% (84-99%)	94% (90-98%)	85% (76-94%)	98% (91-100%)	97% (92-99%)	92% (82-97%)
Samsung Galaxy Watch3	82% (73-92%)	80% (73-86%)	61% (50-72%)	86% (77-95%)	94% (90-98%)	84% (75-94%)	98% (91-100%)	97% (93-99%)	94% (84-98%)

PPV = positive predictive value.

## Discussion

This large, prospective diagnostic study aimed to determine the number of inconclusive SL-ECG tracings and the accuracy of detecting AF when comparing the manufacturers’ algorithms to a novel AI-based cross-platform algorithm offering agnostic interpretation of SL-ECGs among 4 different smart devices. The main findings are as follows: First, 14%–17% of SL-ECGs were rated inconclusive by the manufacturers’ algorithm, regardless of manufacturer and device used for recording. Clearly, improved algorithms are needed to reduce the current burden created by these smart devices on the healthcare system. Second, the novel AI algorithm reduced the rate of inconclusive SL-ECG significantly, to <1%–2% per manufacturer; and overall, only 10 SL-ECGs (1.2%) were deemed inconclusive. Third, inconclusive SL-ECG diagnosis could be reduced while maintaining sensitivity and improving the specificity compared to the manufacturers’ algorithms. Finally, the diagnostic performance of the agnostic AI algorithm was in the range of manual interpretation, thus setting an example for the possibilities of today’s technological capabilities and possible benefits of a deep neural network–based solution for automated rhythm classification and thus allowing an early triage of SL-ECGs.

The reasons for the high rate of inconclusive SL-ECGs by the manufacturers’ algorithm are often unknown, as the algorithms are proprietary. It is crucial to highlight that cardiologists could interpret the smart device ECGs with an impressive success rate of 99%, which is a compelling indication of the fundamental failures within these algorithms in interpreting primary rhythm. Among the leading causes of inconclusive ECG recordings, several factors can be identified. First, a low heart rate, defined as less than 50 beats per minute, can contribute to the inconclusiveness of an ECG reading. Similarly, a high heart rate exceeding 100–150 beats per minute can also challenge obtaining conclusive results. Moreover, noise, whether from external sources or from device-related artifacts, can significantly impact the accuracy and interpretability of ECG readings. Such noise interference can obscure vital signals and introduce errors in algorithmic analysis, leading to inconclusive outcomes. Furthermore, various arrhythmias, including premature atrial contractions and premature ventricular contractions, can contribute to inconclusive ECGs. These irregular heart rhythms can pose challenges for automated algorithms to assess and classify, resulting in inconclusive interpretations.^14^ Nevertheless, there was no systematic issue with the SL-ECGs, as highlighted in Table 2, since in only 1 case all 4 manufacturers’ algorithms reported an inconclusive tracing. Through the recognition of common reasons for inconclusive ECG recordings, healthcare professionals and algorithm developers can work collaboratively to address and rectify the algorithmic failures and facilitate a powerful diagnostic support tool, reducing the overall workload generated through this young (mostly) patient-initiated heart rhythm analysis.

The results of our study extend and corroborate previous work with smart devices^9^^,^^10^^,^^17^^,^^18^ as well as neural networks^19^^,^^20^ and confirm that the amount of inconclusive tracings is higher than initially reported.^21^ New AI or other algorithms seem to offer a possible solution for this issue.^10^^,^^22^ Loop and Holter ECGs are already analyzed for AF episodes by AI algorithms^6^, ^7^, ^8^ and have demonstrated that they are highly sensitive to AF but are vulnerable to a high rate of false-positive detections.^23^ Other forms of detection algorithms, such as a deterministic approach for AF diagnosis,^9^^,^^10^ have proven to offer the possibility of achieving a similar accuracy, though only with extended length and mostly in non–consumer electronic devices or wearables. However, to the best of our knowledge, there is a shortage of data based on 30-second SL-ECG recordings for AF diagnosis. Similarly, more and more AI-based solutions are available in other fields of medical diagnostics, such as radiology^24^ and oncology.

This study confirms that the use of AI solutions may be extended, further assessed, and implemented to smart device ECG rhythm classification. The proposed AI-based algorithm could be valuable to assist physicians with managing the large amount of data in the detection of AF using smart devices and will likely alleviate the clinical burden represented by the manual review of smart device SL-ECGs, as mentioned above. Furthermore, although smart devices have been previously compared in their diagnostic abilities,^3^^,^^15^ cross-platform detection of AF based on an agnostic deep neural network–based algorithm was only very sparsely investigated as of today.

### Limitations

It is important to acknowledge that this study has several limitations. First, we did not repeat inconclusive SL-ECGs. This could have led to a higher number of conclusive tracings and therefore more device-based interpretations resulting in either AF or SR instead of inconclusive. However, this mimics normal clinical practice.

Second, for the PDF SL-ECGs to be processable by the novel AI algorithm in this study, the SL-ECGs had to be vectorized on the digital exported PDF, where rasterized PDF exports were not readable by the automated algorithm at that time point. However, this limitation is being addressed by several manufacturers and is expected to be resolved soon.

Finally, we can only assume that manufacturers implement similar (convolutional network–based) algorithms in their proprietary and possibly variable diagnostic applications. Undisclosed manufacturer algorithms, as well as the uncertainty of access to raw data, will continue to pose limitations for further research in this field.

## Conclusion

A novel AI algorithm reduced the rate of inconclusive SL-ECG diagnosis significantly while maintaining sensitivity and improving the specificity compared to the manufacturers’ algorithms.
